# Application of Headspace Solid-Phase Microextraction for Determination of Chloro-Organic Compounds in Sewage Samples

**DOI:** 10.1080/15376510701624084

**Published:** 2008-06-23

**Authors:** Grażyna Wejnerowska, Jerzy Gaca

**Affiliations:** Chair of Chemistry and Environmental Protection, Faculty of Chemical Technology and Engineering, University of Technology and Agriculture, Seminaryjna 3 St. 85-326, Bydgoszcz, Poland

**Keywords:** Environmental Analysis, Halogenated Compounds, Solid Phase Microextraction, Water Analysis

## Abstract

Solid phase microextraction (SPME) has been optimized and applied to the determination of the volatile halogenated compounds (VHCs) and semi-volatile halogenated compounds (SVHCs). Three types of SPME fiber coated with different stationary phases (PDMS–100 μm, CAR/PDMS-75 μm, PDMS/DVB–65 μm) were used to examine their extraction efficiencies for the compounds tested. Experimental parameters such as the selection of SPME coatings, extraction time, and addition of salts were studied. The carboxen-polydimethylsiloxane (CAR/PDMS) fiber appears to be the most suitable for the determination of VHCs. Analytical parameters such as linearity, limit of detection, and precision were also evaluated. Application of ECD detector for the determination of VHCs and SVHCs allows their determination on the low concentration level, ranging from 0.005 to 0.8 μg/L^−1^. The HS-SPME-GC/ECD procedure gave good analytical precision expressed as relative standard deviation (RSD) (ranged from 5.08% to 8.07%) for a concentration level of 5 μg/L^−1^ and good linearity (r^2^ > 0.98) in a wide calibration range. The applied HS-SPME-GC/ECD method was found to be a quick and effective technique for the determination of microtrace amounts of volatile and semi-volatile halogenated compounds in samples containing high amounts of various organic compounds.

## INTRODUCTION

Volatile organic compounds (VOCs) and semi-volatile organic compounds (SVOCs), especially organochlorines, are an important chemical class of pollutants in water (Santos et al. 1996; Lee et al. 1997). The anthropogenic pollution of environmental water goes into the global environment, thus representing significant public health risk. Volatile halogenated compounds (VHCs) have been shown to affect a wide number of biological and environmental systems; they influence various atmospheric processes: some are carcinogens and/or mutagens, while others are persistent and show bioaccumulation effects. In addition, many VHCs and SVHCs exhibit toxic effects on aquatic organisms (Golfinopoulos et al. 1998; Safarova et al. 2004).

Their use as solvents, cleaning agents, propellants, and fuels in a large number of industrial and commercial applications has led to their ubiquitous presence as water pollutants. VHCs are ubiquitous in the marine environment throughout the world. Trace levels of halocarbons have even been found in Antarctic waters and surface snows. Anthropogenic emissions are mainly held responsible for the presence of these compounds in coastal and open seawaters. However, in situ production by macro- and microalgae has been reported to contribute to the local input of VHCs, especially for low-molecular-mass halocarbons (Huybrechts et al. 2000).

Due to these facts, monitoring of VHCs and SVHCs is mandatory. Their analysis has always been a major challenge to environmental chemists. Several authorities, such as the U.S. Environmental Protection Agency (EPA), the European Union (EU), and national agencies, have included VHCs in their priority pollutants list, and methods have been established for analyzing them in water samples of various origins (Silgoner et al. 1997).

Gas chromatography is often used for the analysis of VHCs and SVHCs. The most difficult and time-consuming step in the determination of organic pollutants in environmental samples is extraction of the analytes from the matrix. Because of low concentration levels found in natural waters (ng/L^−1^ to μg/L^−1^), a preconcentration step is necessary prior to analysis and detection (Golfinopoulos et al. 1998; Safarova et al. 2004).

For the determination of VHCs and SVHCs in water, direct aqueous injection of sample, static headspace (HS) techniques (Golfinopoulos et al. 2001; Safarova et al. 2004; Sakata et al. 2004; Gaca and Wejnerowska 2005), dynamic headspace techniques (P&T, stripping) (Ketola et al. 1997; Lee et al. 1997), liquid-liquid extraction (LLE), membrane techniques, solid phase extraction (SPE), solid phase microextraction (SPME), and distillation techniques are used as sample preparation techniques (Arambarri et al. 2004; Cancho et al. 2001; Koziel et al. 2004; Lattuati-Derieux et al. 2004).

Recently, SPME has been shown to be an excellent alternative to the aforementioned techniques. It is a rapid, inexpensive, solvent-free technique for the extraction of organic compounds from aqueous samples. SPME combines sampling and preconcentration into one step and allows direct transfer of the analytes to the chromatographic column through a standard split/splitless injector. The SMPE fiber is a fused-silica needle coated with a stationary phase fitted in a special syringe-type holder for protection and sampling (Santos et al. 1996; Lambropoulou et al. 2002). High sensitivity can be reached by using a thick and selective stationary phase.

Studies on optimization of the SPME method for the determination of VHCs and SVHCs in water samples have been conducted. Temperature effect, absorption time, and the addition of salt were studied. Analytical parameters such as linearity, limit of detection, and precision were also evaluated. Application of the ECD detector for the determination of VHCs and SVHCs allows their determination on the concentration level of ng/L^−1^. The developed procedure was used for the determination of volatile halogenated compounds occurring in sewage sampled from the chemical plant producing epichlorohydrin and other organic compounds. It was found that the HS-SPME technique was a method suitable for a routine environment monitoring.

## EXPERIMENTAL

### Chemicals

The mixture of 14 volatile and semi-volatile halogenated compounds by Supelco (Bellefonte, PA, USA) for EPA method 502.2 was used for optimization studies. The mixture composition and physicochemical parameters of analytes are presented in Table 1. The standard mixture contained 2000 μg/mL^−1^ of each component dissolved in methanol. Sodium chloride was supplied by POCH S.A. (Gliwice, Poland).

**TABLE 1:** Physicochemical properties of VHCs and SVHCs

No. of analyte		b.p. (°C)	M_r_	d (g/mL^−1^)	log *K*_ow_
1	1,1-Dichloropropylene	–	110.9	–	2.53
2	1,2-Dichloroethane	83.5	98.9	1.253	1.83
3	Trichloroethylene	86.7	131.1	1.462	2.47
4	1,2-Dichloropropane	96.8	112.0	1.155	2.25
5	cis-1,3-Dichloropropylene	104.3	110.1	1.220	2.29
6	trans-1,3-Dichloropropylene	112.0	110.1	1.217	2.29
7	1,1,2-Trichloroethane	113.8	133.4	1.441	2.01
8	1,3-Dichloropropane	120.4	112.0	1.188	2.32
9	1,2-Dibromoethane	131.7	187.9	2.170	2.01
10	1,1,1,2-Tetrachloroethane	130.5	167.9	1.553	2.93
11	1,1,2,2-Tetrachloroethane	146.3	167.9	1.595	2.19
12	1,2,3-Trichloropropane	156.0	147.4	1.389	2.50
13	1,2-Dibromo-3-chloropropane	195.0	236.3	2.050	2.68
14	Hexachlorobutadiene	210.0	260.8	1.680	4.72

*K*_ow_, octanol–water coefficient; values taken from ref. www.syrres.com/esc/kowdemo.htm.

Stock standard solution mixture of these compounds was prepared by introducing 10 μL of standard mixture into a calibrated flask of 100 mL and a solution of 200 μg/L^−1^ was obtained for SPME optimization studies. The secondary standard solutions were prepared by dilution with water of the stock solution to give concentrations of 5 to 200 μg/L^−1^. The secondary standard solutions were used for calibration and determination of limits of detection. Solutions were prepared using distilled/deionized water.

Every day, the fresh stock solutions were prepared for the tests.

### SPME Procedure

The SPME device and fiber coatings polydimethylsiloxane (100 μm PDMS), polydimethylsiloxane-divinylbenzene (65 μm PDMS/DVB), and carboxen- polydimethylsiloxane (75 μm CAR/PDMS) were supplied by Supelco (Bellefonte, PA, USA). Before the first use, the fibers were conditioned in the hot port of the gas chromatograph according to the supplier's instructions.

For the extraction, the solutions were placed in 7-mL screw-cap vials equipped with stir bars and fitted with silicone/PTFE septa.

The vials were filled with the studied solution to the top in the case when the SPME technique was applied, whereas the volume of solution was 5 mL for sorption by the HS-SPME technique. During the extraction step, the solution was stirred vigorously at constant speed (1000 rpm) with a magnetic stirrer at a temperature of 40°C for 25 min. After extraction, the fiber was exposed directly to the hot GC injector for analysis. Time of desorption was 2 min. This time was sufficient for a quantitative desorption of all the analytes studied and reinserting the fiber after the run did not show any carry-over.

### Chromatography

Analyses were performed using a Hewlett-Packard 5890 Series II gas chromatograph equipped with an split/splitless injector port and electron-capture detector (ECD) and mass spectrometry detector (MS-HP 5972).

Helium was the carrier gas, with a flow rate of 1.2 mL/min^−1^. HP-1 column (Crosslinked Methyl Siloxane) 40 m × 0.53 mm × 5 μm was used for GC separation. The temperature program used was 50°C for 4 min, 10°C min^−1^ to 250°C for 8 min. Temperature of the ECD detector was 320°C. The split/splitless injection port (split ratio 1:5) temperature was 250°C, with a helium head pressure maintained at 5.0 psi (1 psi = 6894.76 Pa).

The interface temperature to MS was 280°C. The MS detector was operated in scan mode (electron impact at 70 eV, 1000 V).

### Sampling and Storage

The sewages were sampled on the outflow to the municipal sewage treatment plant, which was supplied with municipal, and the averaged and pretreated sewages from the chemical plant. The sewages were sampled into the dark glass bottles of 2 L. The bottles were prepared by washing with soap and water; rinsing with tap water, ultrapure water, and acetone; and placing in an oven at 150°C for 2 h. The bottles were filled to the top and tightly closed. Samples were stored at a temperature of 4°C and they were subjected to analytical procedures on the day of sampling.

## RESULTS AND DISCUSSION

The VHCs and SVHCs contained in the mixture composition were chosen in such a manner that they would be suitable for the determination of the trace amounts of these analytes in surface waters and sewages, since the method was supposed to be used in the region of industrial sector, where the main pollutants were chlorinated aliphatic compounds.

A series of optimization studies of the method was carried out prior to application of the SPME technique for determination of VHCs in environmental samples. Parameters of the SPME technique were chosen to obtain possibly the highest extraction efficiency and precision of results.

The standard mixture, characterized by composition and properties presented in Table 1, was used for the choice of the chromatographic analysis conditions and parameters of extraction on the sorption fiber. Numeration of the individual analytes presented in Table 1 is consequently used in the further part of this paper.

### Selection of Sorption Fiber

Studies on optimization of the SPME technique were begun with the choice of sorption fiber that would allow obtaining the best extraction effects. Three types of fibers with various polarities-polydimethylsiloxane (100 μm PDMS), polydimethylsiloxane-divinylbenzene (65 μm PDMS/DVB), and carboxen-polydimethylsiloxane (75 μm CAR/PDMS)–were applied. The solution of 200 μg/L^−1^ was prepared and analyses by the HS-SPME-GC-FID technique were conducted for each fiber. All the analyses were carried out at constant parameters of procedure: temperature of extraction of 50°C, extraction time of 20 min, stirring at 1000 rpm, temperature of injector 250°C; operating parameters of GC were presented in the Chromatography section.

**FIGURE 1:**
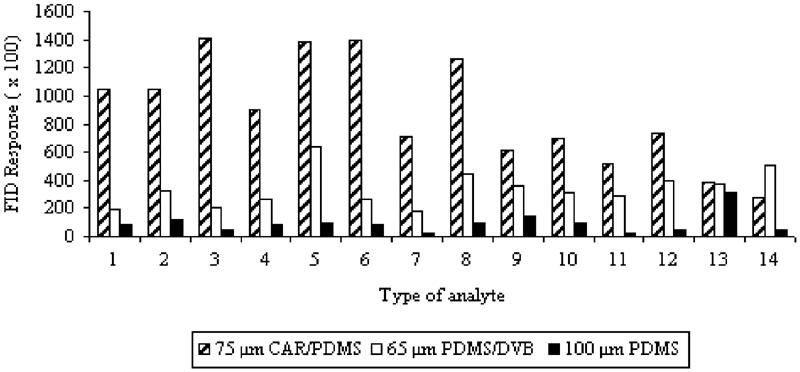
Dependence between peak areas and type of sorption fiber applied for VHC and SVHC mixtures.

Extraction efficiency of the VHC and SVHC mixtures for the individual fibers is presented in Figure 1. The response areas were obtained from the average of three replicates.

It was observed that the type of fiber had a very significant effect on the sorption efficiency of the analyzed VHCs and SVHCs. First of all, the PDMS fiber is usually chosen for the studies of the majority of volatile organic compounds. However, in the case of our mixture of compounds, it showed the lowest sorption efficiency in spite of its high capacity (100 μm). It was certainly caused by its strong nonpolar character.

Our studies showed that the mixed phase coating is more suitable for small molecules (M_r_ < 170). For the majority of analytes (mainly with low molar mass), the 75-μm CAR/PDMS fiber demonstrated considerably better sorption effects. The comparable or even higher sorption efficiency was obtained for 65 μm PDMS/DVB fiber exclusively in the cases of 1,2-dibromo-3-chloropropane and hexachlorobutadiene.

The 75-μm CAR/PDMS fiber that was characterized by the best sorption efficiency for the majority of analytes was selected for the further studies.

### Comparison Between SPME and HS-SPME Techniques

**FIGURE 2:**
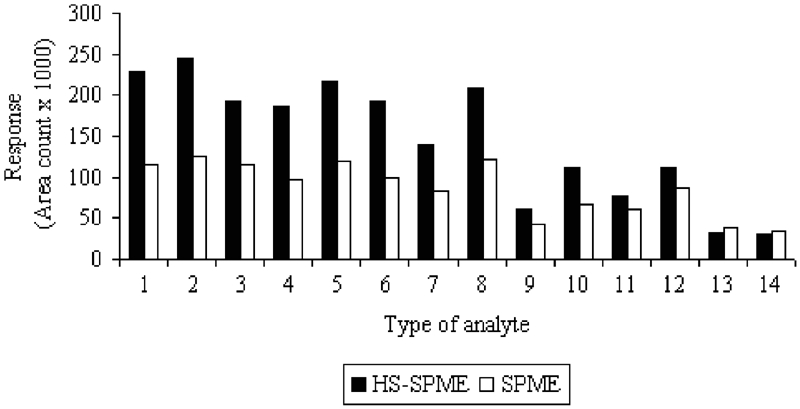
Comparison between the responses obtained by SPME and HS-SPME techniques.

Two methods of fiber exposition were studied. The first one involved immersing (75 μm CAR/PDMS) fiber into the aqueous phase (SPME) and, in the second one, the fiber was suspended in the headspace above the water (HS-SPME). The prepared solution of 200 μg/L^−1^ and parameters defined in the section Selection of sorption fiber were applied in investigations. The results obtained are illustrated in Figure 2.

For the majority of analytes (especially for more volatile ones), the significant differences in detector responses (for concentration of 200 μg/L^−1^) were observed for various methods of fiber exposition. Increase in sorption efficiency for more volatile analytes ranged up to 50%. Only the analytes with higher boiling points (b.p. >130°C) showed lower difference in detector response. Exclusively in the cases of 1,2-dibromo-3-chloropropane and hexachlorobutadiene (b.p. >190°C), the slight increase in sorption efficiency was observed for the SPME technique.

Taking into account the fact that sampling by headspace technique will be mainly applied for the analysis of environmental samples with rich matrixes, the obtained results are satisfying. This technique allows the selective isolation of volatile and semi-volatile halogenated compounds with simultaneous elimination of the matrix effects and prolongation of the sorption fiber life.

### Effect of Fiber Exposition Time on Sorption Efficiency

Time of the fiber exposition (i.e., the time required to reach interfacial equilibrium) is essential to obtain maximum efficiency, precision, and low limits of detection.

**FIGURE 3:**
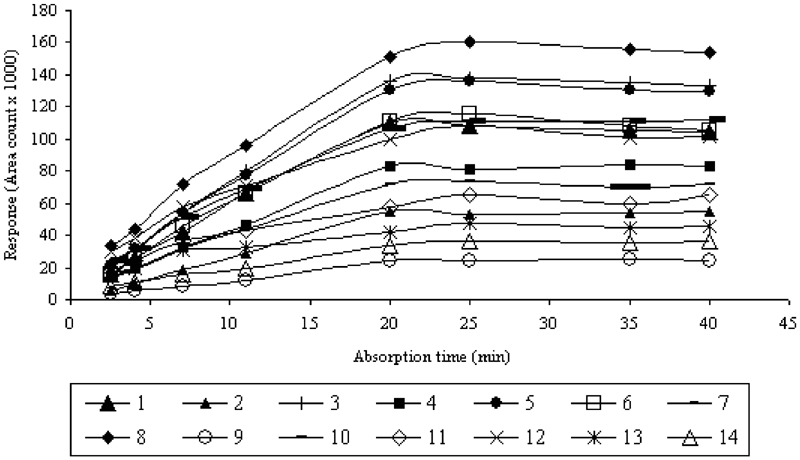
Dependence between extraction time and detector response for VHCs and SVHCs at concentration of 200 μg/L^−1^.

Stirring of the sample reduced the time needed to reach equilibrium because it enhanced the diffusion of analytes toward the fiber. The stirring speed of the aqueous solution during the HS-SPME procedure was optimized. At stirring speed of 1000 rpm, the equilibrium time was reached after 20 to 25 min, whereas using lower speeds a time of 40 to 45 min was needed. Figure 3 shows the example of the adsorption time profiles for VHCs and SVHCs at a concentration level of 200 μg/L^−1^ in water using the 75-μm CAR/PDMS fiber.

As is shown in Figure 3, the increase in extraction efficiency was observed during the first 20 min. It can be concluded that the equilibrium is reached between 20 and 25 min for all the components. Time equal to 25 min is the optimum one for all the VHCs and SVHCs analyzed by the HS-SPME technique.

### Effect of Temperature on Extraction Efficiency

The extraction temperature has two opposing effects on the SPME process. An increase in temperature during extraction enhances the diffusion of analytes toward the fiber, decreasing the time needed to reach the equilibrium. Moreover, in the HS-SPME sampling mode, the temperature helps transfer analytes to the headspace. On the other hand, this increase in temperature reduced the distribution constant of the analytes because the absorption step was an exothermic process (Peňalver et al. 1999).

**FIGURE 4:**
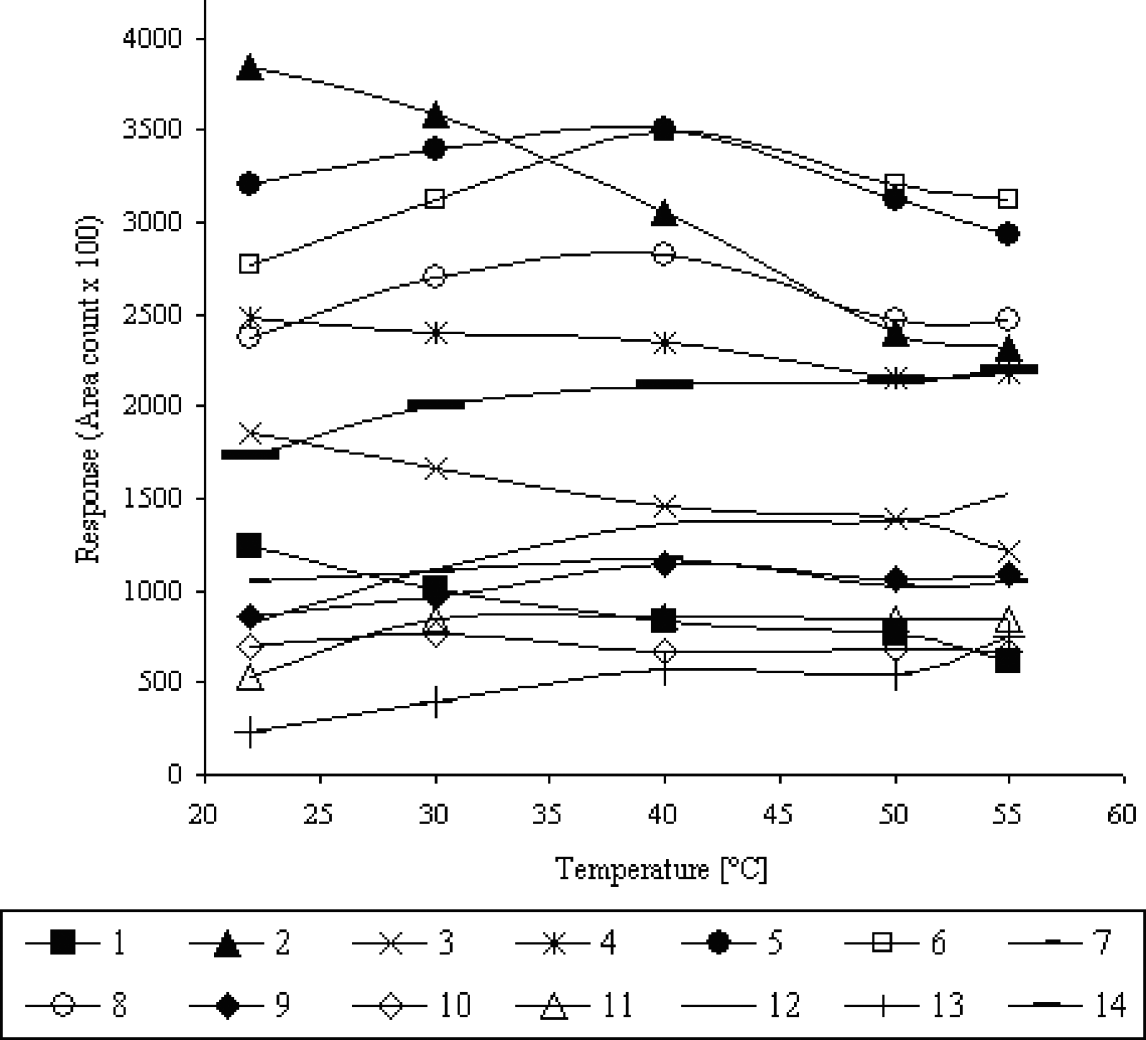
Effect of temperature on the absorption of VHCs and SVHCs.

In order to examine the effect of temperature on VHC and SVHC sorption efficiency, the solution of 200 μg/L^−1^ was prepared and CAR/PDMS fiber was used. The extraction (HS-SPME) was conducted during 25 min and temperature of process was changed within the range from 20°C to 55°C. The obtained results are presented in Figure 4.

For the mixture of 14 compounds, the various effect of temperature rise on the absorption process was observed. In the case of analytes nos. 1 to 4 (b.p. < 100°C), the sorption efficiency decreased as the temperature of extraction was raised. However, analytes with a boiling point from 100°C to 130°C showed the increase in sorption efficiency as the temperature of solution was raised to 40°C and a further temperature rise resulted in a decrease of sorption efficiency. The distinct increase in extraction efficiency with the rise of temperature was observed for semi-volatile compounds (nos. 11–14) with boiling points higher than 130°C.

Due to the various effects of temperature on extraction efficiency for so many analytes, the peak areas of all analytes were summarized and it was found that 40°C was a compromise temperature of extraction.

### Effect of the Sample Salting out on Extraction Efficiency

The effect of the salt addition to the water samples was studied. It is well known that sorption processes are affected by ionic strength of the sample, which is adjusted by addition of salt (Lambropoulou et al., 2002). For this purpose, water samples spiked with VHC and SVHC mixtures were extracted and analyzed after addition of different amounts of sodium chloride in the range from 0% to 30% (w/v). Effect of salting out was studied by applying the HS-SPME technique. Because the SPME extraction is conducted by headspace instead of direct immersion in a concentrated salt solution, the problem due to salt crystallization on the fiber after a hot injection, which occasionally contributes to premature breakage of the fiber, can be avoided (Fang et al. 2003).

It was observed that salting out of the sample had an insignificant effect on extraction efficiency (HS-SPME). Some analytes showed a slight increase in the degree of extraction; however, in the case of other ones, a slight decrease was observed. A slight (i.e., 4% to 5%, summarized all peak areas) increase in the amount of analytes absorbed on fiber was achieved by addition of 20% to 30% of NaCl. Therefore, the sample salting out was abandoned in subsequent studies.

### Linear Range, Limits of Detection, and Precision

The parameters defined for SPME and HS-SPME procedures (in the section SPME procedure) were used for statistical evaluation of the methods and the limits of detection were determined for the individual analytes using FID and ECD detectors (Table 2). In order to access linearity of the HS-SPME method, the calibration studies were carried out by preparing the standard mixture at six concentration levels. The linear range for each analyte is listed in Table 2. All the compounds studied were characterized by correlation coefficients (r^2^) better than 0.98.

**TABLE 2:** Limits of detection, linearity, and precision of HS-SPME method

	GC-FID				GC-ECD			
		
No. of analyte	Correlation coefficient (r^2^)	Linear range (μg/L^−1^)	LOD (μg/L^−1^)	RSD (%) (n = 6)	Correlation coefficient (r^2^)	Linear range (μg/L^−1^)	LOD (μg/L^−1^)	RSD (%) (n = 6)
1	0.998	10–200	5.4	6.65	0.989	1.0–100	0.4	6.32
2	0.984	20–200	9.5	7.52	0.982	1.0–100	0.4	4.40
3	0.997	20–200	6.0	4.71	0.983	0.1–100	0.005	5.21
4	0.993	10–200	3.3	5.82	0.984	0.1–100	0.06	5.08
5	0.991	20–200	7.8	6.88	0.989	1.0–100	0.3	7.25
6	0.989	20–200	7.8	7.26	0.989	1.0–100	0.8	7.12
7	0.980	20–200	7.8	6.92	0.988	0.5–100	0.10	5.88
8	0.991	20–200	7.8	4.88	0.983	1.0–100	0.4	6.51
9	0.985	20–200	10.0	5.63	0.997	1.0–100	0.2	6.66
10	0.991	20–200	5.0	5.44	0.989	1.0–100	0.8	5.88
11	0.992	20–200	7.8	3.22	0.987	0.1–100	0.06	5.80
12	0.990	5–200	1.2	4.20	0.981	0.5–100	0.01	8.07
13	0.998	5–200	1.0	6.50	0.997	0.5–100	0.08	6.66
14	0.987	10–200	4.5	4.54	0.987	0.5–100	0.12	5.23

The precision of the HS-SPME method for the FID detector was evaluated for a concentration level of 50 μg/L^−1^ to give a relative standard deviation (RSD) between 3.22% and 7.52%. The precision of the HS-SPME for the ECD detector was evaluated for a concentration level of 5 μg/L^−1^ to give an RSD between 4.40% and 8.07% (Table 2). The number of replicates was six. These values show that the methods are precise.

The sensitivity of the techniques was considered in terms of limits of detection (LODs), which depended on the method and the instrument sensitivity.

The LOD was defined as the concentration of an analyte that produced a signal three times greater than the baseline noise. The average signal to noise of five replicates at low concentrations was used to calculate the LOD (Table 2). Limits of detection for all the VHCs and SVHCs determined by FID detector were on the level of μg/L^−1^. Application of the ECD detector allowed reduction of limits of detection by 10 to 1000 times and made possible to determine some of them on the level of ng/L^−1^.

### Analysis of Sewage Samples

The developed procedure for the determination of VHCs and SVHCs by the SPME technique was applied for quantitative and qualitative analysis of the selected analytes in sewage samples. Waste waters were sampled from the municipal sewage treatment plant supplied with sewages from chemical plants producing epichlorohydrin, nitro-compounds, dyes, and other organic compounds. In order to assess the usability of the developed procedure for the determination of the low contents of chloro-organic compounds, the purified waste waters were sampled on exit from the sewage treatment plant.

The preliminary investigations comprised studies on the effect of the sampling method (SPME, HS-SPME) on sorption efficiency of the individual compounds.

**FIGURE 5:**
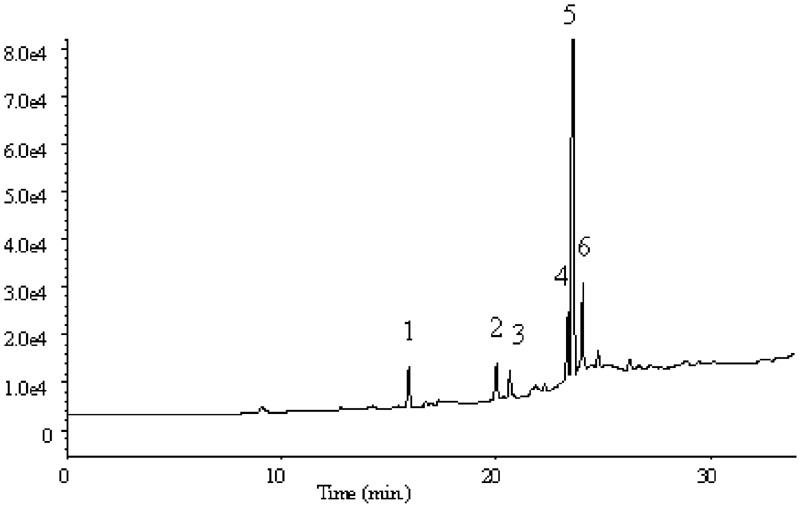
Chromatogram obtained as a result of sewage analysis by HS-SPME-GC/MS method.

The analyses carried out by applying the FID detector showed no differences between quantities and qualities of analytes determined by these techniques (SPME, HS-SPME). However, taking into account the fact that various organic substances that are present in real water (humic and fulvic acids) are also present in sewage samples, we decided to apply the HS-SPME technique in further studies with the aim to avoid a premature destruction of fiber. Studies were repeated by making preliminary qualitative analyses using the MS detector. The chromatogram obtained as a result of analysis is presented in Figure 5.

As a result of GC-MS analysis, the following compounds were identified: (1) phenol, (2) 1,2,3-trichloropropane, (3) 1,4-dinitrobenzene, (4) 1-methyl-4-nitrobenzene, (5) 2-methyl-1,3-dinitrobenzene, and (6) 1,3,5-trinitro-2-methylbenzene.

**FIGURE 6:**
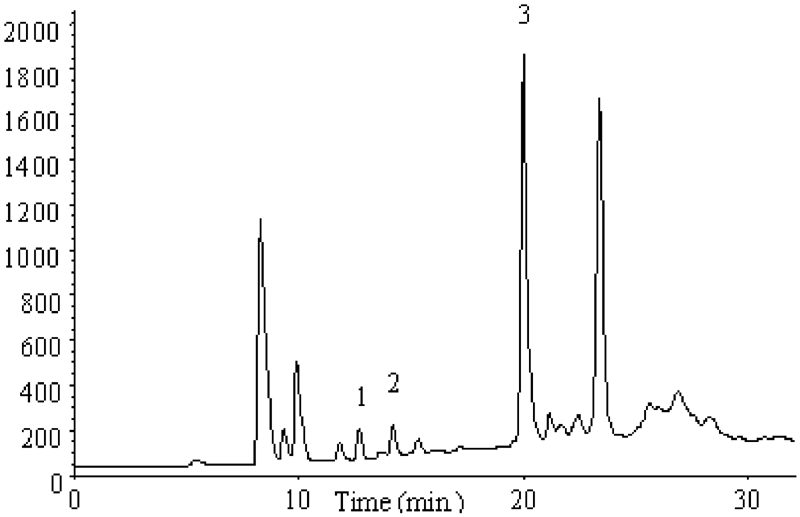
Chromatogram obtained as a result of sewage analysis by HS-SPME-GC/ECD method.

Analyses with application of FID and MS detectors showed 1,2,3-trichloropropane and trace amounts of volatile compounds. Further studies were carried out by the HS-SPME technique with the ECD detector, which was considerably more sensitive to chloro-organic compounds. The chromatogram obtained is presented in Figure 6.

The qualitative identification of analytes was performed (Fig. 6) by applying standard solutions of compounds suspected to be present in target samples. Identification of analytes was performed by comparing the retention times of the individual compounds and by internal standard addition. Three chloro-organic compounds, which were the subject of our studies, were identified in sewage samples. The identified analytes were as follows: (1) 1,2-dichloropropane, (2) 1,3-dichloropropane, and (3) 1,2,3-trichloropropane.

The precision of technique was assessed by analysis of sewages in eight replicates and then recording the peak areas of the target compounds. The RSD was calculated and its value ranged from 6.6% to 11.5% for three compounds studied.

Quantitative analyses were performed by standard addition; the increasing amounts of compounds were introduced into sewage samples and their content in sample was read out from the drawn calibrating curves. The following contents of analytes were determined: 1,2-dichloropropane, 0.72 μg/L^−1^; 1,3-dichloropropane, 3.80 μg/L^−1^; and 1,2,3-trichloropropane, 13.2 μg/L^−1^.

In order to check the effect of matrix on extraction efficiency, the consecutive studies consisting in determination of analyte recoveries from sewage samples were carried out. The standard mixture of analytes was introduced into the sewage sample to obtain a concentration of 20 μg/L^−1^ for each analyte. Recoveries for sewage sample with target compounds were obtained by interpolating in the HS-SPME calibration curves. In the case of three analyzed compounds, the results were corrected with the determined quantities. For all the compounds studied, the recoveries were determined and they ranged from 96% to 102%.

SPME proves to be the competitive technique in comparison with other commonly applied methods of sample preparation for analysis (HS, LLE, SPME, P&T) since it allows the determination of low concentrations in sewage samples, and moreover, it is characterized by a high precision and simple procedure. As regards the VHC analysis, the SPME method has a significant advantage–it is solvent free, which eliminates coincidence of solvent with analytes.

## CONCLUSIONS

The investigated SPME procedure is a fast, inexpensive, and solvent-free method that has been proved to be precise and sensitive for analysis of volatile and semi-volatile halogenated compounds in water and sewage samples. Mixture of 14 halogenated (b.p. ∼80–210°C) were studied and the best results for all compounds were obtained while applying a 75-μm CAR/PDMS fiber.

The HS-SPME-GC/ECD technique is characterized both by good precision and a good linearity within the wide range of concentrations. Detection limits in the ng/L^−1^ level were obtained. The present study has shown that the optimized HS-SPME procedure can be proposed as a fast monitoring method for the analysis of volatile halogenated compounds in the industrial sewages.
